# Primary culture of germ cells that portray stem cell characteristics and recipient preparation for autologous transplantation in the rhesus monkey

**DOI:** 10.1111/jcmm.17197

**Published:** 2022-02-01

**Authors:** Huaqin Yuan, Jiachen Sun, Shengnan Wang, Ziyi Xiang, Fan Yang, Yaping Yan, Yanchao Duan, Lufan Li, Xin Wu, Wei Si

**Affiliations:** ^1^ Cancer Center of Nanjing GaoChun People’s Hospital Nanjing China; ^2^ State Key Laboratory of Reproductive Medicine Nanjing Medical University Nanjing China; ^3^ State Key Laboratory of Primate Biomedical Research Institute of Primate Translational Medicine Kunming University of Science and Technology Kunming Yunnan China

**Keywords:** germ cell transplantation, in vitro culture, Rhesus monkey, spermatogonia, spermatogonial stem cells

## Abstract

Fertility preservation for prepubertal cancer patients prior to oncologic treatment is an emerging issue, and non‐human primates are considered to constitute suitable models due to the limited availability of human testicular tissues. However, the feasibility of spermatogonial stem cell (SSC) propagation in vitro and autologous testicular germ cell transplantation *in vivo* requires further exploration in monkeys. Herein, we characterized germ cells in macaque testes at 6 months (M), 18 M and 60 M of age, and effectively isolated the spermatogenic cells (including the spermatogonia) from macaque testes with high purity (over 80%) using combined approaches of STA‐PUT separation, Percoll gradients and differential plating. We also generated recipient monkey testes with ablated endogenous spermatogenesis using the alkylating agent busulfan in six macaques, and successfully mimicked autologous cell transplantation in the testes under ultrasonographic guidance. The use of trypan blue led to successful intratubular injection in 4 of 4 testes. Although SSCs in culture showed no significant propagation, we were able to maintain monkey testicular germ cells with stem cell characteristics for up to 3 weeks. Collectively, these data provided meaningful information for future fertility preservation and SSC studies on both non‐human primates and humans.

## 1 INTRODUCTION

Cancer treatments in prepubertal boys can produce long‐term damage to their testicular microenvironment and can extend into adulthood, as the gonadotoxicity precipitated by chemotherapy is principally attributed to the administration of alkylating agents that are a mainstay of clinical treatments.^1^ Cryopreservation of spermatozoa is a common strategy of fertility preservation for adult males, but it is not an option for prepubertal boys who are not yet capable of producing mature sperm.^2^ Currently, the most favourable fertility preservation strategy is the cryopreservation of immature testicular biopsy, since the biopsy pieces containing spermatogonial stem cells (SSCs) could restore fertility in the future through tissue grafting or in vitro spermatogenesis.^3^ Following the successful transplantation of SSCs and subsequent fertility restoration in mice,^4^, ^5^ many similar attempts have been made in other large animal species over recent decades.^6^, ^7^, ^8^ However, novel methods are now under investigation for establishing a culture system that expands the human SSC population in vitro, and that allows transplantation to the testes to be more feasible and efficient following cancer treatment and cure.^9^, ^10^, ^11^

SSCs possess the properties of self‐renewal and differentiation that allow them to maintain the stem cell pool while continuously producing sperm throughout adult life.^12^ The development of SSCs into spermatozoa (termed spermatogenesis) is a strictly regulated process that involves the mitosis of spermatogonia, meiotic division of spermatocytes, and spermiogenesis.^13^ Studies of SSC biology in rodents have recently significantly improved our understanding of the mechanisms underlying spermatogenesis^14^, ^15^ owing to the achievements of *in vivo* and in vitro methods and techniques of SSC manipulation; these include the establishment of long‐term SSC culture systems.^16^ and SSC transplantation technology.^4^ However, these methods and techniques in large animals—including human and primates—remain relatively limited. Spermatogenesis is, however, highly conserved in mammals.

It has been shown that gene activity in mouse gonocytes (the precursor cells of SSCs) and prepubertal human spermatogonia shares great similarity.^15^ There are also many disparities in the spermatogenic lineages between primates and rodents, as the two species diverged phylogenetically at least 75 million years ago.^17^ including the classification of germ cell subtypes and the kinetics of spermatogenesis.^18^ Since non‐human primates (NHPs)—in particular the rhesus monkey—are highly comparable to humans in terms of testicular physiology, NHPs have been applied as the most suitable preclinical models for human germ‐cell transplantation.^19^

Several approaches have been developed for the characterization of germ cell populations—including magnetic‐activated cell sorting (MACS),^20^ fluorescence‐activated cell sorting (FACS) with fluorescent dyes,^21^, ^22^ and STA‐PUT velocity sedimentation,^23^ and these represent currently popular methodologies to separate various testicular cells. Cell sorting using FACS has been successfully performed for the isolation of highly purified germ cells in multiple species.^24^ The STA‐PUT method—while unsuitable for the enrichment of spermatocytes and spermatids due to the lack of established surface biomarkers in primates—holds several advantages over FACS, including larger yields of cells per testis and higher cell viability, thus making the isolated cells obtained from STA‐PUT more feasible for culture.^25^ Using STA‐PUT, research groups have achieved the successful isolation of spermatogenic cells with high purity and viability in mice,^23^ cattle^26^ and humans.^27^ And these cells can be used for numerous analyses—including changes in gene expression,^28^ nucleosomal dynamics,^29^ chromatin remodeling,^30^ and other dynamic aspects of germ cells during spermatogenesis such as non‐coding RNAs and the use of protein profiling.^31^, ^32^, ^33^

In the present study, we reported our approaches to purifying testicular germ cells in rhesus monkeys by STA‐PUT, and also demonstrated the primary culture of germ cells with stem cell characteristics—as well as recipient preparation and attempts at testicular transplantation of germ cells using the rhesus monkey model.

## 2 MATERIALS AND METHODS

### 2.1 Animals

All experiments involving macaques (*Macaca mulatta*) were approved by the Institutional Animal Care and Use Committees (IACUC, authorization code: LPBR202104015) of Kunming University of Science and Technology. All experiments were performed in accordance with the institutional guidelines of Kunming University of Science and Technology, as appropriate.

### 2.2 Ultrasound‐guided injections of the rete testis

All ultrasonographic measurements were performed with a 12.0 MHz linear superficial probe. Each monkey was anaesthetized and placed on an operating table, lying on its back, and ultrasound transmission gel was applied to its scrotum. The rete testis showed high echogenicity under ultrasonographic examination and could therefore be visualized from systematic longitudinal and transverse scans. The injection was performed from the lower part of the testis under continuous ultrasonographic monitoring, requiring approximately 30 min. Finally, the injected testes (*n* = 4) were collected and further bisected to evaluate the effect of transplantation.

### 2.3 Testis histology and immunofluorescence

For histology, testicular tissues were surgically removed and fixed with Hartman's Fixative (Cat#H0290‐500ML, Sigma, USA) for 24 h at room temperature. Tissues were dehydrated, embedded in paraffin and sectioned at 5 μm. Sections were then de‐paraffinized and stained with haematoxylin (Cat#HHS16, Sigma) and eosin (Cat# E607321‐0100, Sangon Biotech) (H&E). For tissue immunofluorescence, frozen testis sections were prepared and washed three times with PBS, and blocked with 1% BSA and 0.1% Triton X‐100 for 1 h at room temperature. Slides were incubated with primary antibodies directed against UCHL1 (PGP9.5, 1:200, Cat#UC1‐H5140, AbD Serotec), γH2AX (Cat#16‐202A, Merck & Millipore) and FITC‐PNA (Cat#L7381, Sigma) diluted in 1% BSA. Following overnight incubation, slides were washed three times with PBS and incubated with secondary antibodies (1:100) and Hoechst staining solution for 1 h at room temperature. Images were collected via light microscopy (ZEISS, LSM700, Germany).

For cellular immunofluorescence, cells were fixed with 2% PFA (Cat#P6148, Sigma) at 4°C overnight, and after washing 3 times with PBS, slides were blocked with 10% goat serum containing Triton X‐100 for 1 h at room temperature. Slides were then washed with PBS and incubated with primary antibodies for 2 h and secondary antibodies for 1 h in the dark. Images were collected through a Zeiss microscope as described above.

### 2.4 Preparation of testicular cell suspensions

Testis tissues from each rhesus monkey were minced and digested with Enzyme Solution I (20 ml of DMEM [Cat#11965‐084, Gibco], 80 mg of collagenase IV [Cat#C5138, Sigma], 20 mg of DNase I [Cat#DN25, Sigma], 50 mg of hyaluronidase [Cat#H3884, Sigma], and 200 μl of penicillin‐streptomycin [Cat#15140‐122, Gibco]) for 30 min at 37°C. Cells were centrifuged at 600 × g for 5 min at 4°C, and the supernatant was discarded. We then incubated the cells with Enzyme Solution II (20 ml of 0.25% trypsin‐EDTA [Cat#25200‐114, Gibco], 80 mg of collagenase IV [Cat#C5138, Sigma], 20 mg of DNase I [Cat#DN25, Sigma], 50 mg of hyaluronidase [Cat#H3884, Sigma], and 200 μl of penicillin‐streptomycin [Cat#15140‐122, Gibco]) for 15–20 min (5‐year‐old monkey) or 28 min (6‐M‐old monkeys) at 37°C. We filtered the cell suspension with a 100‐μm strainer following FBS addition, and 2 ml of red blood cell lysis buffer was added and the suspension incubated for 5 min at 4°C. After filtration through a 40‐μm strainer, germ cells were resuspended in 25 ml of wash buffer (50 ml of HBSS [Cat#14175‐079, Gibco], 50 mg of DNase I [Cat#DN25, Sigma], and 500 μl of penicillin‐streptomycin [Cat#15140‐122, Gibco]) for use in the STA‐PUT method.

### 2.5 STA‐PUT

**FIGURE 1:**
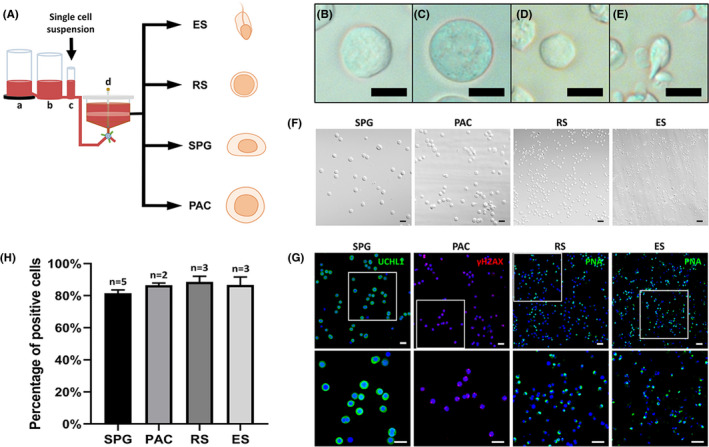
Germ cell isolation from monkey testes and purity verification. (A) Experimental flow diagram of the STA‐PUT apparatus. Separation of germ cells was conducted using the STA‐PUT gravity sedimentation method, with linear BSA gradients generated by the medium mixture in three glass containers, from left to right: (a) a chamber with 4% BSA medium, (b) a chamber with 2% BSA medium and (c) a chamber with loading cell suspension in 0.5% BSA medium. The cells were then separated by gravity in the sedimentation chamber (d). (B–E) Morphology of isolated cells as observed under phase‐contrast microscopy, showing spermatogonia, pachytene spermatocytes, round spermatids and elongated spermatids. (F) Phenotypic characteristics and (G) immunofluorescence identification of cells isolated by STA‐PUT. Cells with identical morphology were observed with DIC microscopy, and germ cells were identified by UCHL1 as spermatogonia (green), γH2AX as pachytene spermatocytes (red), and PNA as round or elongated spermatids (green). DAPI was applied as a nuclear stain. (G) The lower panels show higher magnifications of the white boxes in the upper panels. (H) We then quantified purity of the separated cells. Data represent the mean of the value ± 1 standard deviation, and two to five separate experiments were conducted (scale bars, 10 μm [B–E]; 20 μm [F‐G])

STA‐PUT was performed as described previously with some modifications.^25^ Testes from 2 adult (60‐M‐old monkey, 60 M) macaques were used for separation of pachytene spermatocytes, round spermatids and elongated spermatids, and testes from 5 neonatal macaques (6‐M‐old monkey, 6 M) were used for separation of spermatogonia. We added 800 ml (in the case of the 60‐M‐old rhesus monkey) of DMEM containing 4% BSA into chamber a, and 800 ml (60‐M‐old monkey) or 300 ml (6‐M‐old monkey) of DMEM containing 2% BSA into chamber b (Figure 1A). Germ cells (in total of 5 × 10^8^ cells for 800 ml and 1 × 10^8^ cells for 300 ml) were resuspended in 25 ml of wash buffer and allowed to slowly flow into the sedimentation chamber after addition to the cell chamber. When the cells were all in the sedimentation chamber, we opened the clamps to let 2% and 4% BSA flow into the sedimentation chamber at a rate of 45 ml min^−1^. Once the BSA was in the sedimentation chamber, we allowed the cells to sediment for 3 h (60‐M‐old monkey) or 1.5 h (6‐M monkey). We collected cells at a rate of 10 ml/48 s, and each collection tube was filled with 10 ml of cell suspension.

### 2.6 Purification of spermatogonia

The spermatogonial population that we collected from STA‐PUT was resuspended in 2 ml of DMEM. A gradient of Percoll was then prepared in a 15‐ml tube by consecutively adding 0.5 ml of 45% Percoll, 2 ml of 35% Percoll, and 2 ml of 20% Percoll. We slowly added the cell suspension along the wall of the tube to the Percoll gradient, and centrifuged it at 600 g for 20 min at 4°C. We subsequently collected the phase with enriched spermatogonia and repeated the Percoll purification.

### 2.7 Culture of spermatogonia

Testes from 5 neonatal (6 M) macaques were used for cell culture study. The testicular single‐cell suspension was inoculated on the 0.1% gelatin‐coated wells of a 6‐well plate in fibroblast culture medium supplemented with bFGF (1 ng ml^−1^). Adherent cells were collected after 48 h of plating and treated with mitomycin C to create feeder cells. Spermatogonial culture was then performed as described previously with some modifications.^34^ Thirty percent Percoll was used to remove the dead cells and debris from the monkey testicular cells. Then, testicular cells were resuspended in fibroblast cell medium (450 ml of DMEM [Cat#11965‐084, Gibco], 50 ml of fetal bovine serum [Cat#10099‐141, Gibco], 5 ml of L‐glutamine [Cat#25030‐081, Gibco], 5 ml of penicillin‐streptomycin [Cat#15140‐122, Gibco], and 3.6 μl of 2‐mercaptoethanol) and plated onto 0.1% gelatin‐coated plates. After a 48‐h incubation, adherent cells were collected and treated with 10 μg ml^−1^. mitomycin C (Sigma, M4287) for 3 h as feeder cells, and the floating germ cells were resuspended in serum‐free mouse medium and plated onto the feeder layer. Germ cells were maintained at 37°C in a humidified atmosphere containing 5% CO_2_ in compressed air with 20 ng ml^−1^. glial cell line‐derived neurotrophic factor (GDNF, Cat# 212‐GD‐050/CF, R&D), 1 ng ml^−1^ basic fibroblast growth factor (bFGF, Cat# 233‐FB‐025/CF, R&D), 100 ng ml^−1^ GDNF family receptor alpha‐1 (GFR 1, 560‐GR‐100/CF, R&D), 20 ng ml^−1^ epidermal growth factor (EGF, Cat#SRP3196, Sigma), 0.05 ng μl^−1^ bone morphogenetic protein 7 (BMP7, Cat#120‐03P, PeproTech), and 104 U ml^−1^ leukaemia inhibitory factor (LIF, 11668030, Invitrogen).

### 2.8 Establishment of transplantation platform

Recipient preparation was performed as described previously.^6^ Briefly, adult rhesus monkeys (*n* = 6) were administered granulocyte colony‐stimulating factor (G‐CSF, Amgen) at a dosage of 20 μg/kg/day for 6 days by subcutaneous injection. Twenty‐four hours after the collection of peripheral blood stem cells (PBSCs), rhesus monkeys were given 12 mg kg^−1^ of busulfan intravenously (Busulfex IV, PDL BioPharma), and 18 h later PBSCs were administered subcutaneously. Two days later, G‐CSF (300 μg kg^−1^) was injected intravenously into the rhesus monkeys. Trypan blue transplantation was performed 10–12 weeks after busulfan treatment, and 1 ml of contrast medium and 0.7 ml of trypan blue solution were injected into the tubules of the monkey testes.

### 2.9 qPCR analysis

The cultured cells originally from one of the neonatal (6 M) monkey testis isolation were used for qPCR analysis. Total RNA was extracted from cell culture wells. The reverse transcription was performed using a PrimeScript RT reagent kit (Cat#DRR036A, Takara). qPCR was then performed using SYBR Premix Ex Taq II (Cat#DRR820A, Takara). Gene primers are shown in Table 1.

**TABLE 1:** Primer sequences of genes used for RT‐PCR

Genes		Primers (5’−3’)
ACTIN	Forward Reverse	CCACCATGTACCCTGGCATT AGGGCCAGACTCGTCATACT
DAZL	Forward Reverse	AACTTACATGCAGCCCCCAA CAGCTGAATAAGCCGGAGGT
UTF1	Forward Reverse	GCGACATCGCGAACATCCT AGGGACACTGTCTGGTCGAA
FGFR3	Forward Reverse	CTCGGGAGATGACGAAGACG TGCCATTCTTCAGCCAGGAG
VIMENTIN	Forward Reverse	AATGGCTCGTCACCTTCG AGTTTCGTTGATAACCTGTCC
GATA4	Forward Reverse	GGAAGCCCAAGAACCTGAATAA GCTGTGCCCGTAGTGAGATGA
ZBTB16	Forward Reverse	TTGAGCATGCCATCTTCGGT TATCAGGAAGCTCGACCCCA

## 3 RESULTS

### 3.1 Characterization of germ cells in monkey testes

**FIGURE 2:**
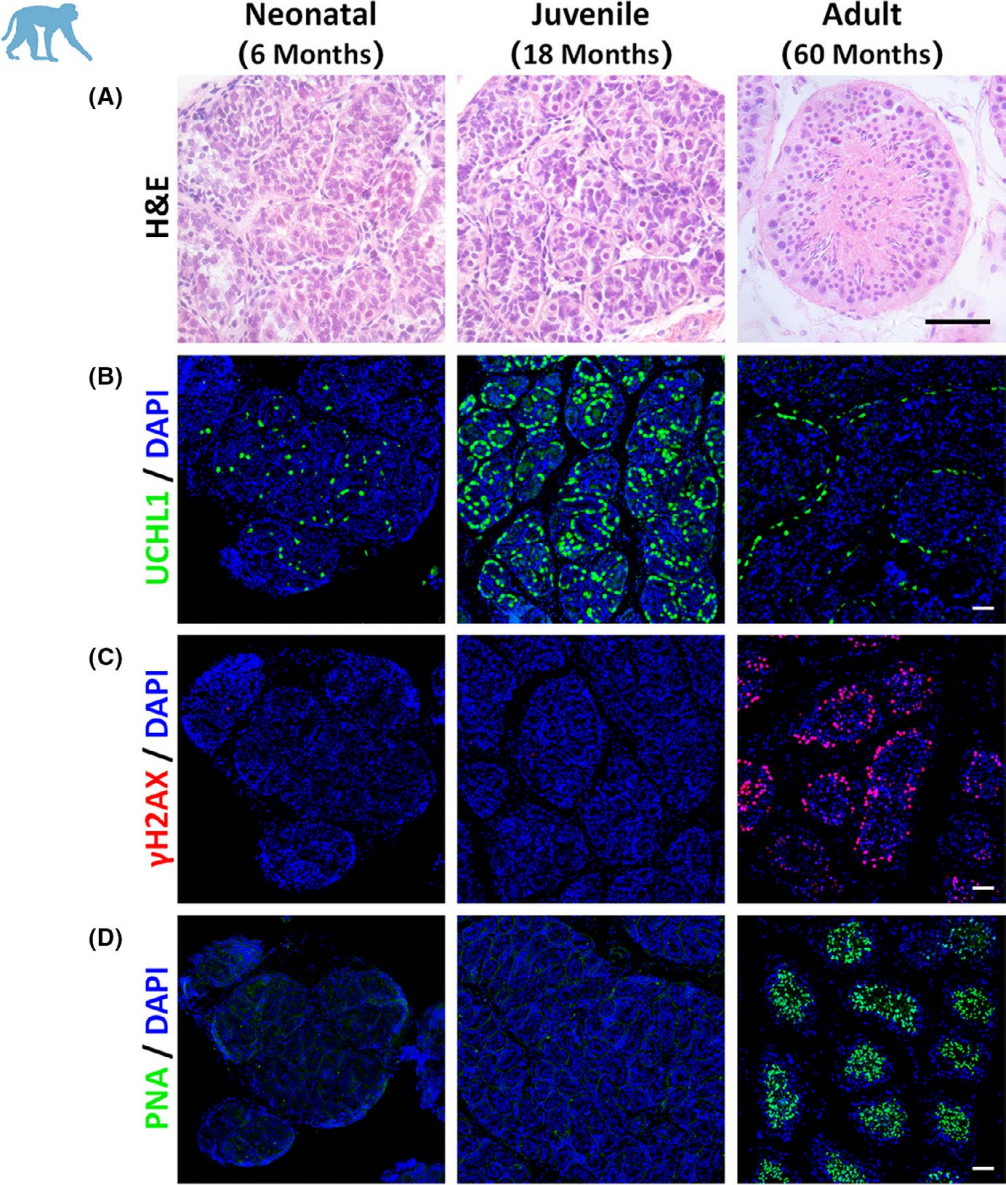
Germ cell characterization in testes from monkeys at different ages. (A) Seminiferous tubules are shown in neonatal (6 M), juvenile (18 M), and adult (60 M) rhesus monkey testes. The histologic morphology of seminiferous tubules was demonstrated by H&E staining. (B–D) Germ cells in the testes were identified by UCHL1 marker as spermatogonia (B, green), by γH2AX as pachytene spermatocytes (C, red), and with PNA as spermatids (D, green). DAPI was applied as a nuclear stain (scale bars, 50 μm)

To discern this, we selected monkeys at three ages: neonatal (6 months of age, 6 M), juvenile (18 M) and adult (60 M), so as to represent various levels of reproductive maturity for the phenotypic characterization of the testes (Figure 2A). We first explored the histology of the testes using H&E staining and showed that neonatal (6 M) and juvenile monkeys (18 M) had not yet initiated spermatogenesis and lacked late‐stage germ cells and spermatozoa. However, we observed regular spermatogenic epithelial waves in the seminiferous tubules in the adult testis (60 M). We next used immunofluorescence staining to confirm monkey germ cell development in the testes. UCHL1 (ubiquitin carboxyl‐terminal hydrolase isozyme L1, also called PGP 9.5), which is predominantly localized to the cellular cytoplasm, is a well‐established marker for undifferentiated spermatogonia in multiple species.^35^, ^36^ As shown in Figure 2B, UCHL1 marked spermatogonia along the basement membrane of the seminiferous tubules in testes from monkeys at all three ages. We demonstrated that the phosphorylated form of H2AX (γH2AX)—a marker for DNA double‐strand breaks that is generally accepted in the identification of pachytene spermatocytes in the testes^37^—was expressed in the seminiferous tubules of 60‐M‐old monkey testes (Figure 2C), but that it was not observed in either neonatal or juvenile monkey testes; this suggested to us that the middle or late stages of spermatogenesis (including meiosis) were not initiated by 18 months after birth. Since PNA (peanut agglutinin) exclusively binds to the outer acrosomal membrane of spermatids as a marker for spermatids,^38^ we next demonstrated notable expression of PNA only around the lumen of the seminiferous tubules in the adult testes (Figure 2D). Control stainings for immunofluorescence analysis were shown in Figure S1. We also characterized the germ cells at the developmental stages of testes following chronologic growth in monkeys; specifically, that germ cells in testes from monkeys at 6 M and 18 M of age had not yet entered meiosis, confirming the presence of undifferentiated or differentiating spermatogonia. In contrast, the seminiferous tubules of adult testes exhibited complete spermatogenesis with spermatogenic cells at all stages. These data provided the information needed for proper timing to determine which chronologic age would be feasible for the separation of spermatogenic cells from the testes.

### 3.2 Separation of spermatogenic cells with the STA‐PUT method

Using our knowledge of germ cell composition at different testicular maturational stages in rhesus monkeys, we generated the approaches necessary to isolate and purify the subtypes of germ cells according to donor ages. In particular, testes from neonatal (6‐M) monkeys were selected to isolate spermatogonia (diploid germ cells, 2N), while adult monkeys were used to isolate pachytene spermatocytes (tetraploid, 4N), and round spermatids and elongated spermatids (haploid, 1N). First, testicular single‐cell suspensions were generated from a two‐step enzymatic digestion and subjected to the STA‐PUT separator (see Methods for details, Figure 1A). After sedimentation by gravity, consecutive droplets of separated germ cells were collected in 15‐ml conical tubes. We subsequently identified by phase‐contrast microscopy and a micrometre reticle in the eyepiece for the diameter and morphologic characteristics of the cells in each tube. Spermatogonia (SPG) ranged in diameter from 14 to 16 μm (Figure 1B), pachytene spermatocytes (PAC) were between 16 and 18 μm (Figure 1C), round spermatid (RS) diameters were approximately 10 μm and characterized by large nuclei (Figure 1D), and elongated spermatids (ES) were oval in shape with protruding tails (Figure 1E). Pooled cells with preferentially enriched spermatogonia and pachytene spermatocytes that were collected according to the majority size distribution were further purified through a Percoll gradient (see Methods). The viability of all cell types obtained was above 95% as evaluated by trypan blue exclusion. Next, we examined the isolated cells by differential interference contrast (DIC) microscopy (Figure 1F) and immunostaining (Figure 1G) for verification of purity. Gross cellular morphology appeared to be identical by DIC microscopy, and we further confirmed the purity using marker proteins as shown in Figure 1G. SPG was defined by the expression of UCHL1, and PAC was identified by γH2AX. Both RS and ES were confirmed by PNA staining on the acrosomes (Figure 1G). In general, the purity of harvested SPG was 81.6+/‐1.8%, PAC was 86.6+/‐1.0%, RS was 88.6+/‐2.9%, and ES was 86.7%+‐4.0% using our present approaches Figure 1H.

### 3.3 Culture of monkey germ cells with stem cell characteristics

**FIGURE 3:**
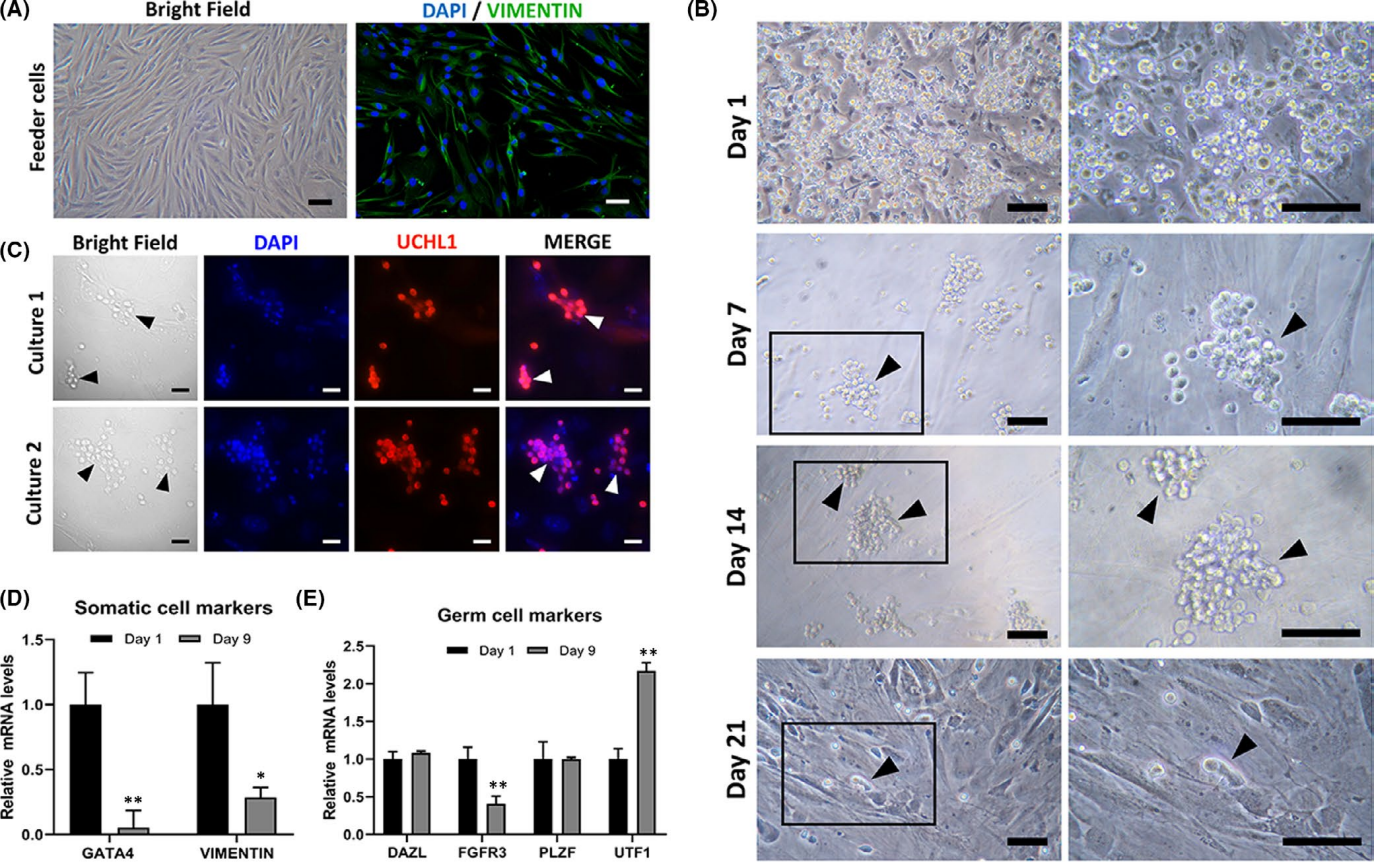
Primary culture of monkey testicular cells with stem cell characteristics"(A)Fibroblasts isolated from adult monkey testicular cells by differential plating are shown, and their purity was validated by vimentin staining (green). The cells were then treated with mitomycin C as feeder layers for germ cell culture. (B) We observed the morphology of cultured germ cells from initial plating (day 1) until 3 weeks (day 21) in a defined serum‐free medium supplemented with growth factors (see Methods). Grape‐shaped cellular clumps were observed in the culture plates, as shown by arrows. The right panels show higher magnifications of the black boxes in the left panels (except for the first row). (C) Immunofluorescence of UCHL1 staining is shown to verify germ cells. Cells cultured for 9 days were analysed from two independent experiments. DAPI staining was applied as a nuclear marker. Real‐time quantitative reverse‐transcription PCR was applied to analyse the relative expression of somatic cell (D) and germ cell markers (E) on day 0 and day 9, respectively. Data represent means ± 1 SEM from 3 independent experiments (^*^ *p* < 0.05 or ^**^ *p* < 0.01 [Student's t test], scale bars, 50 μm [A‐B]; 20 μm [C])

Long‐term SSC propagation in vitro has been successfully achieved in rodents; however, reproducible culture systems in large animals are still under investigation. We thus explored primary cultures of monkey germ cells following the separation of spermatogonia in testes from neonatal monkeys. Testes from 5 neonatal (6 M) macaques were used for the cell culture study. We first generated a testis fibroblast feeder layer by differential plating with medium containing 7% fetal bovine serum. The identity of fibroblast cells was confirmed by cellular morphology and staining with the intermediate filament protein vimentin, a marker for testicular somatic cells (Figure 3A). Control staining for immunofluorescence analysis was shown in Figure S1. When cell growth reached approximately 90% confluency in wells of the plates, the fibroblasts were treated with mitomycin C to inactivate cell division before applying them to germ cell culture as the feeder layer (Figure 3A). Testis germ cells were harvested from neonatal monkey testes (representing UCHL1‐positive spermatogonia in vivo) and plated on 0.1% gelatin‐coated culture wells for 24 h to remove possibly adherent cells. Cells were then subjected to further culture on the fibroblast feeder layer with a defined serum‐free SSC culture medium supplemented with a combination of the growth factors GDNF, GFR alpha1, and FGF2 in the medium, as previously applied to rodent SSC culture.^14^, ^39^ Identical to initial cultures of mouse or rat SSCs, germ cells on the plates formed the typical grape‐like cell clumps in the wells, and these clumps could be successfully maintained for 14 days after initial seeding (Figure 3B). However, the clumps gradually detached from the feeders and were often lost by medium changes at intervals of 2–3 days, and both the size and number of clumps were significantly decreased following 21 days in culture medium (Figure 3B), even when a few cell clumps were still found after 3 weeks of culture.

To characterize the cultured cells, we collected an aliquot of cells cultured for 9 days from two independent culture experiments and validated them by UCHL1 immunofluorescence. The expression of UCHL1 in the clumps demonstrated that there was spermatogonial aggregation (Figure 3C). Next, we analysed the expression of somatic cell markers and germ cell marker genes in cells on days 1 and 9 of culture respectively. The expression levels of *Gata4* and *Vimentin*, marker genes for somatic cells, were significantly decreased by 9‐day culture (Figure 3D). When we analysed the expression levels of *Dazl* (which is deleted in azoospermia and is a germ cell marker gene) and *Zbtb16* (an undifferentiated‐spermatogonia marker gene) on day 9, we found that they were comparable to the levels on day 1. In contrast, *Fgfr3* (fibroblast growth factor receptor 3), a marker gene that reflects the stem cell status of spermatogonia in humans,^40^, ^41^ was significantly reduced, and *Utf1* (undifferentiated embryonic cell transcription factor 1), a marker gene that reflects SSC differentiation status in humans,^42^ was significantly upregulated by our 9‐day culture (Figure 3E).

### 3.4 Ablation of endogenous spermatogenesis in testes of recipient monkeys

**FIGURE 4:**
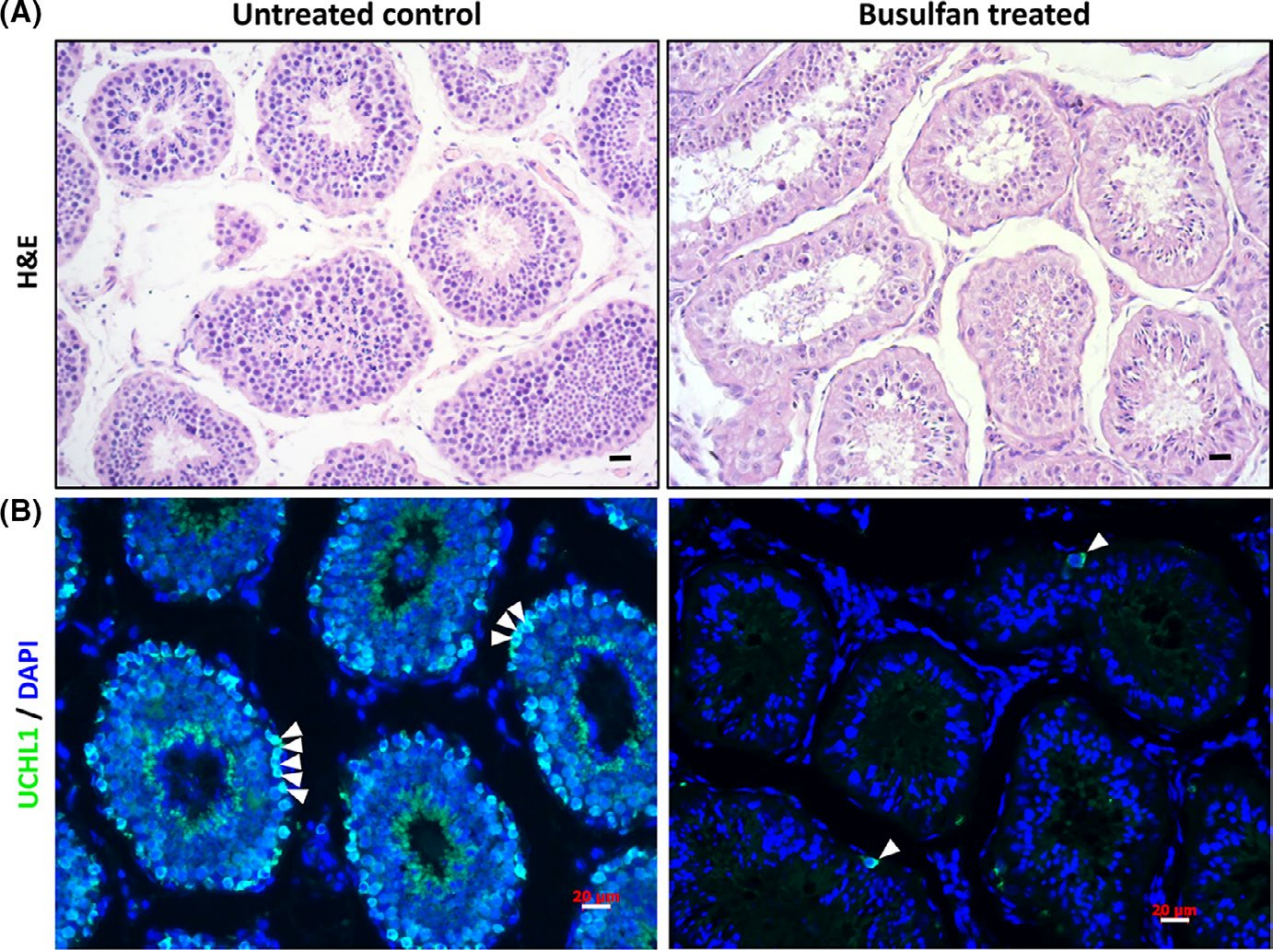
Evaluation of seminiferous tubules in monkey testes using busulfan treatment (A) In the 60‐M‐old monkey testes, histologic analysis indicated that dramatic degeneration of spermatogenic epithelium took place in the seminiferous tubules 12 weeks after busulfan treatment (right) in comparison with the normal monkey testes (left). (B) A majority of the UCHL1‐positive spermatogonia (green) were eliminated 12 weeks after busulfan treatment (right). Cell nuclei were stained by DAPI, and staining was performed on adult rhesus testes (scale bars, 20 μm)

Most previous large‐animal models of transplantation have entailed the use of radiation to deplete endogenous spermatogenesis,^43^, ^44^, ^45^ as considerable injury to the haematopoietic system is provoked by the toxicity of alkylating agents; however, alkylsulfonate busulfan is favourable to the establishment of testicular recipient models for cellular transplantation in mice^46^ and monkeys.^47^ We therefore utilized the approach reported by Hermann et al. to eliminate endogenous spermatogenesis while simultaneously counteracting the damage to the hematopoietic system. Six monkeys were treated with the chemotherapeutic agent busulfan, and after busulfan treatment, prophylactic pre‐stored autologous blood transfusions were conducted (see Methods and Figure 4A). Twelve weeks after busulfan treatment, the testes of adult rhesus monkeys were surgically removed and analysed in histologic sections. Unlike the untreated control testes with intact spermatogenic epithelium, substantial loss and degeneration of spermatogenic cells in the seminiferous tubules were found in the busulfan‐treated monkey testes (Figure 4A). We further validated the spermatogonia (including SSCs) by immunofluorescence staining of UCHL1 and showed the apparent diminution of UCHL1‐positive cells in the basal membrane of seminiferous tubules in the busulfan‐treated monkey testes, even when few spermatogonia remained relative to the untreated testes (Figure 4B). This evidence suggested that a majority of spermatogonia—including SSCs—were successfully depleted by busulfan treatment in monkey testes.

### 3.5 Ultrasound‐guided transplantation into recipient monkey testes

**FIGURE 5:**
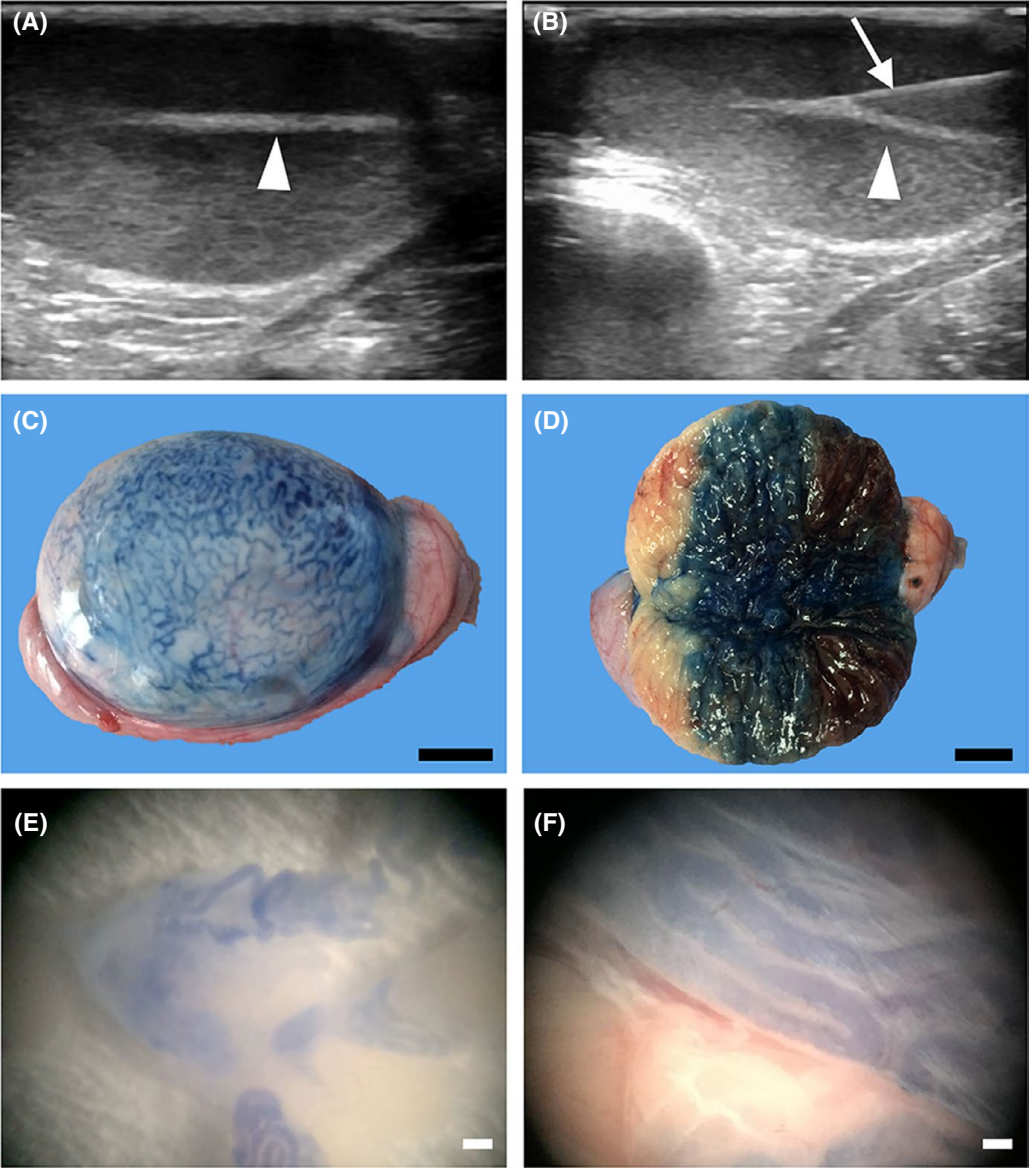
Ultrasound‐guided injection of the rete testis (A) Saline solution with trypan blue (approximately 1 ml) was introduced into the recipient's seminiferous tubules through testicular rete injection. The rete testis of a 60‐M‐old rhesus monkey (arrowhead) is visible under ultrasonographic guidance. Videos are presented in our Supporting Data. (B) An injection needle (arrow) was used to puncture the rete testis, and the solution was gently injected. (C) Trypan blue‐stained seminiferous tubules can be observed on the surface of the injected testis. (D) Bisection of the injected testis indicated that ultrasound‐guided injection was not invasive. The lumen of the testicular seminiferous tubules was filled with trypan blue to approximately 70%–80% of the testicular volume. (E and F) The presence of trypan blue solution in the lumen of seminiferous tubules was assessed through a stereoscope after dissociating testicular tubules in a culture dish, thus confirming successful intratubular injection (scale bars, 1 cm in C and D; 500 μm in E; and 200 μm in F)

In contrast to mouse SSC transplantation in which surgery is typical, we employed the ultrasound‐guided rete testis‐injection method pioneered by Schlatt and colleagues, which generates less physical damage (with reduced invasiveness) and greater efficiency in cell injection.^45^ To pave a platform for the SSC transplantation to rhesus monkey recipients, we explored the feasibility of an injection of trypan‐blue saline solution into the seminiferous tubules by following the protocols reported by Hermann et al.^6^ Under the guidance of ultrasonographic imaging, the injection needle was observed to puncture through the scrotal skin and enter the rete testis (Figure 5A), where all the seminiferous tubules converge in the testis of the recipient monkey. After confirming that the injection needle had been placed in the rete testis by administering the ultrasound contrast agent (Figure 5B and the Supplementary Online Video 1), we then changed the injection solution to a trypan‐blue saline solution (Figure 5B). We allowed approximately 1 ml of blue dye solution to permeate the testis through the testicular ducts, and the injected testis was harvested by surgical removal for subsequent gross and microscopic inspections. The presence of blue dye in the lumen of the seminiferous tubules suggested that approximately 70% of the testicular area was covered by the dye after the administration of a total of 1 ml of saline solution (Figure 5C‐D). In addition, the visible blue dye in the lumen of the seminiferous tubules further suggested the successful injection of saline solution into the seminiferous tubules of the monkey testis (Figure 5E‐F). These data showed our attempts at autologous transplantation of testicular germ cells in primates to rescue fertility in males.

## 4 DISCUSSION

In recent decades, much progress has been made in the study of spermatogonial biology in rodents. However, little is known about the isolation of germ cell subtypes and technical feasibility of SSC transplantation in primates. Here, by our knowledge, we are the first time to isolate spermatogenic cells from rhesus monkey testes and collect the representative cell populations—including spermatogonia, pachytene spermatocytes, round spermatids and elongated spermatozoa with high purity and viability. These isolated monkey germ cells with typical grape‐like cell clumps were maintained for 21 days using a modified SSC culture system, which was previously applied in rodents. Moreover, the feasibility of primate SSC transplantation technique was demonstrated by the successful ultrasound‐guided injection of Trypan blue into the seminiferous tubules. These evidences together have provided meaningful information for future fertility preservation and SSC studies on both non‐human primates and humans.

NHPs, especially rhesus monkeys, are the most suitable preclinical model for clinical translation because of their high similarity to humans in genome as well as testicular structure and physiology.^18^ Therefore, it is necessary to establish the methods for the isolation of spermatogenic cells of multiple stages from monkey testes, and developing such methods should allow us to answer questions regarding primate spermatogenesis; for example, dissecting the chromatin dynamics and molecular reprogramming of chromatin structure from diploid to haploid cells.^30^ The STA‐PUT method has been applied to the isolation of mouse testis cells^31^ and has also been implemented in the isolation of human testicular cells.^48^ While we showed that the yield from adult monkey testes of pachytene spermatocytes and other late‐stage spermatids was of high purity, we did not achieve the same result with spermatogonia because of their similarity in size to somatic cells and other uncharacterized germ cells. Magnetic separation (MACS) was reported to be one way to isolate spermatogonial populations (including SSCs) in mice and humans via conjugation to highly specific antibodies against a particular surface antigen such as CD90.2^49^ or GPR125.^50^ Unfortunately, neither marker was applicable to spermatogonial isolation from rhesus monkey testes in our previous attempts; nonetheless, a combination of STA‐PUT, Percoll‐gradient separation and differential plating significantly improved the purity of monkey spermatogonia from the testes of a 6‐M‐old prepubertal male (Figure 1H).

The recovery of SSCs from prepubertal human testes via clinical biopsy samples alone is unlikely to prove satisfactory for the restoration of fertility following autologous transplantation since the number of Ad and Ap spermatogonia with stem cell potential in this population is likely to be extremely low.^18^, ^51^ Thus, culture and expansion in the number of SSCs are essential for the effective clinical application of human SSC transplantation to restore fertility. Moreover, to reverse patient infertility caused by genetic defects may largely necessitate an improvement in genetic modification modalities or correction through SSCs. For example, exogenous gene integration or successful infertility rescues have been achieved in mice.^52^, ^53^, ^54^ In the past few decades, many attempts have been undertaken to establish long‐term culture of higher primate (nonhuman and human) SSCs,^10^ while protocol feasibility has been rarely established, and existing primate SSC culture methods under various conditions were maintained for a short time (less than 2 weeks) in other reports.^55^, ^56^ In the present study, we also explored the possibility of in vitro culture of germ cells with stem cell identities. We were able to maintain these clump‐forming germ cells for 21 days, and the cultured germ cells were positive for the undifferentiated spermatogonial stem cell and germ cell markers FGFR3, UTF1, DAZL and ZBTB16, which was identical to the data reported by Sharma and colleagues.^57^ However, significant cellular proliferation did not occur following culture (Figure 4). We strongly suggest that the prerequisites of mouse SSC culture are beneficial for the periodic maintenance of primate SSCs, but the defined conditions for the propagation of primate SSCs remain arcane. Our present results are consistent with previous in vivo results with respect to primate (including human) SSC fates after transplantation into the testes of mouse recipients.^6^ The successful culture of monkey SSCs may be determined by three critical aspects, the initial enrichment of SSCs, the medium supplemented with essential growth factors and the trophic feeder layers. Optimization of these aspects might help successful primate SSC propagation in vitro. Removing testicular somatic cells may be a key point for SSC culture because these cells could outgrowth in the culture and stimulate germ cell differentiation including SSCs.^58^, ^59^ The enrichment of germ cells can be significantly improved by differential plating with the combination of gelatin and laminin coating, for example, increasing germ cell purity by 2.7‐fold.^55^ Moreover, the enriched germ cells can be further purified after plating through magnetic microbeads conjugated with anti‐GFRA1 antibody,^56^ since GFRA1 is a surface marker for undifferentiated spermatogonia reported to be expressed in multiple species. Further attempts to test the best combinations of growth factors on primate SSCs are required for self‐renewal and proliferation of SSCs. For example, FGF9, as a novel extrinsic growth factor, was recently reported to promote mouse SSC propagation,^60^ while its effect on primate SSC has not been evaluated. In addition, the stemness of mouse SSCs were found to be significantly improved by altering culture conditions that favour glycolysis as the primary bioenergetics process.^61^ The glycolysis‐optimized conditions were achieved by removal of lipids and free fatty acids from the medium and reduced oxygen tension (10%), where self‐renewal of mouse SSCs were reported to be enhanced via glycolysis mediated by MYC/MYCN.^62^ Overall, the findings of optimized mouse SSC culture conditions would provide great insight into the improvement culture of primate SSCs, however; this needs to be investigated further in future studies.

Prepubertal testicular tissue has recently been demonstrated by the Orwig lab to mature and produce functional sperm when the tissue was autologously grafted under the skin of castrated pubertal rhesus macaques.^43^ The same laboratory also showed successful autologous SSC transplantation that produced spermatogenesis by busulfan‐treated macaques.^47^ These advances suggested that improvements in transplantation methods and well‐established recipient preparation for autologous transplantation using primate models are required for future preclinical trials of human germ cell transplantation and fertility rescue. We followed the protocols used by Orwig and associates and evaluated germ cell deletion in the recipient testes of 60‐M‐old macaques treated with busulfan, and found that endogenous spermatogenesis was successfully depleted, as demonstrated by degenerated seminiferous tubules; however, we also (rarely) observed UCHL1‐positive spermatogonia in the seminiferous tubules of monkey testes 12 weeks after treatment, which demonstrated reliable recipient preparation for cell transplantation. In addition, the successful intra‐testicular cellular transplantation in monkeys needs to be completed under the guidance of ultrasonography, which is slightly different from mouse testicular cell‐transplantation technology. Nevertheless, our approach has been proven feasible and reliable using testes from adult monkeys treated for 12 weeks with busulfan.

To mimic the clinic steps for fertility recovery, two prerequisites are not well achieved. Firstly, genetic modification is not yet feasible, while the second concern is the surgical procedure. In the present study, we have provided information on the developmental characterization of germ cells in the testes, the culture of spermatogonia, recipient preparation, and transplantation trials in the rhesus monkey. Collectively, these are constructive data that will allow the further study of SSCs in primates.

## CONFLICT OF INTEREST

The authors declared no potential conflicts of interest.

## AUTHOR CONTRIBUTION

**Huaqin Yuan:** Data curation (equal); Investigation (equal). **Jiachen Sun:** Data curation (equal); Writing – original draft (lead); Writing – review & editing (lead). **Shengnan Wang:** Data curation (equal); Resources (equal). **Ziyi Xiang:** Investigation (equal). **Fan Yang:** Writing – original draft (supporting). **Yaping Yan:** Investigation (supporting). **Yanchao Duan:** Investigation (supporting). **Lufan Li:** Validation (lead); Writing – review & editing (equal). **Wei Si:** Resources (lead). **Xin Wu:** Writing – review & editing (lead).

## AUTHOR CONTRIBUTIONS

X.W and W.S involved in conception and design, final approval of manuscript, provided administrative and financial support; H.Y, J.S, S.W, Z.X, Y. Y, Y. D and L.L involved in collection and/or assembly of data; J.S, L.L, X.W involved in data analysis and interpretation; J.S, F.Y, L.L, X.W and W.S involved in manuscript writing;

## Supporting information

Supplementary Material

Supplementary Material

Supplementary Material

Supplementary Material

Supplementary Material

Supplementary Material

Supplementary Material

Supplementary Material

Supplementary Material

Supplementary Material

## Data Availability

The data that support the findings of this study are available from the corresponding author upon reasonable request.
