# Supplementary figures and images for: Primary culture of germ cells that portray stem cell characteristics and recipient preparation for autologous transplantation in the rhesus monkey
Source: J Cell Mol Med. 2022 Feb 1;26(5):1567–78. doi: 10.1111/jcmm.17197 (PMC8899175; doi:10.1111/jcmm.17197)

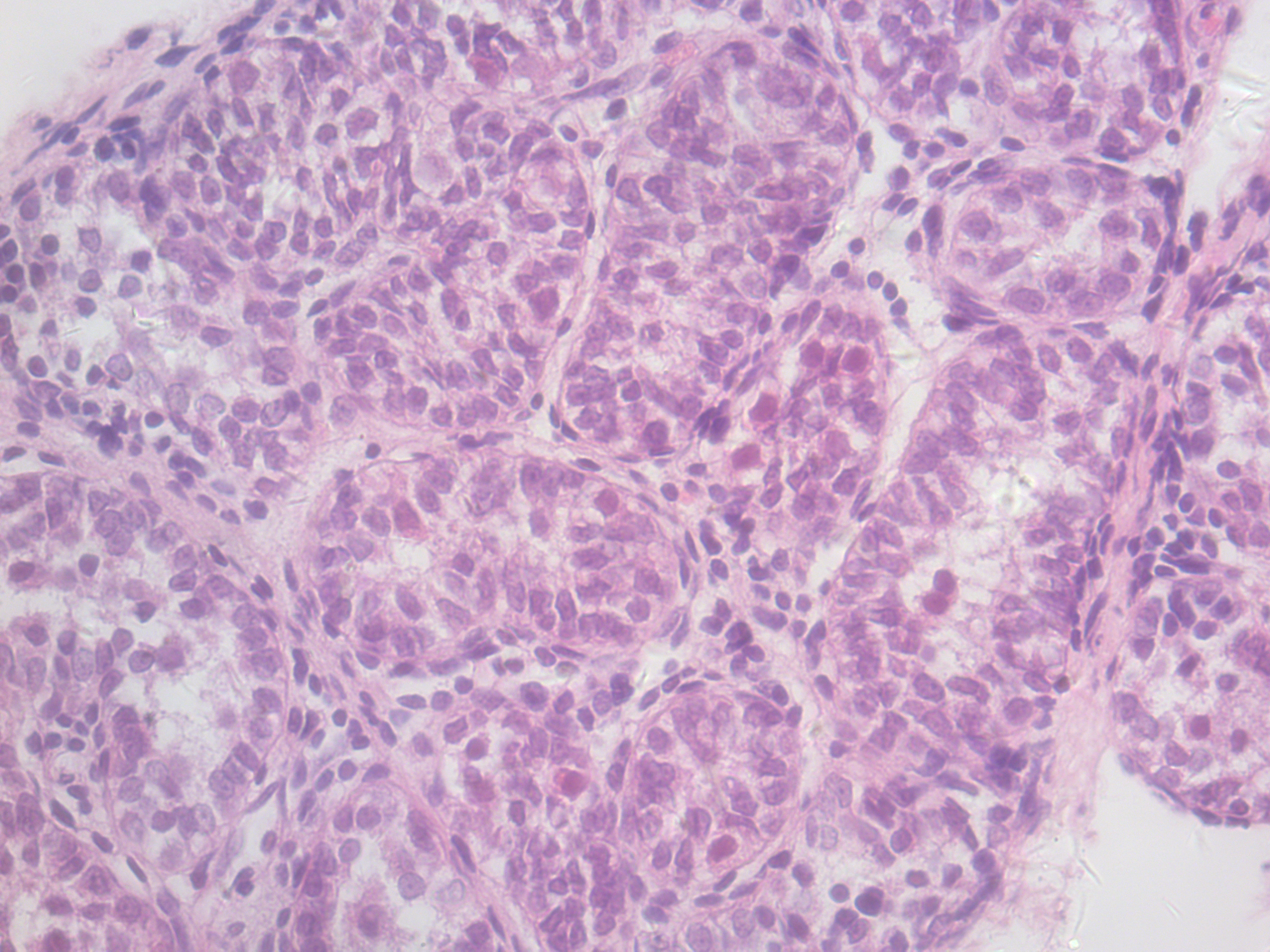

Supplement: Supplementary file 1 — Supplementary Material [file JCMM-26-1567-s005.tif]

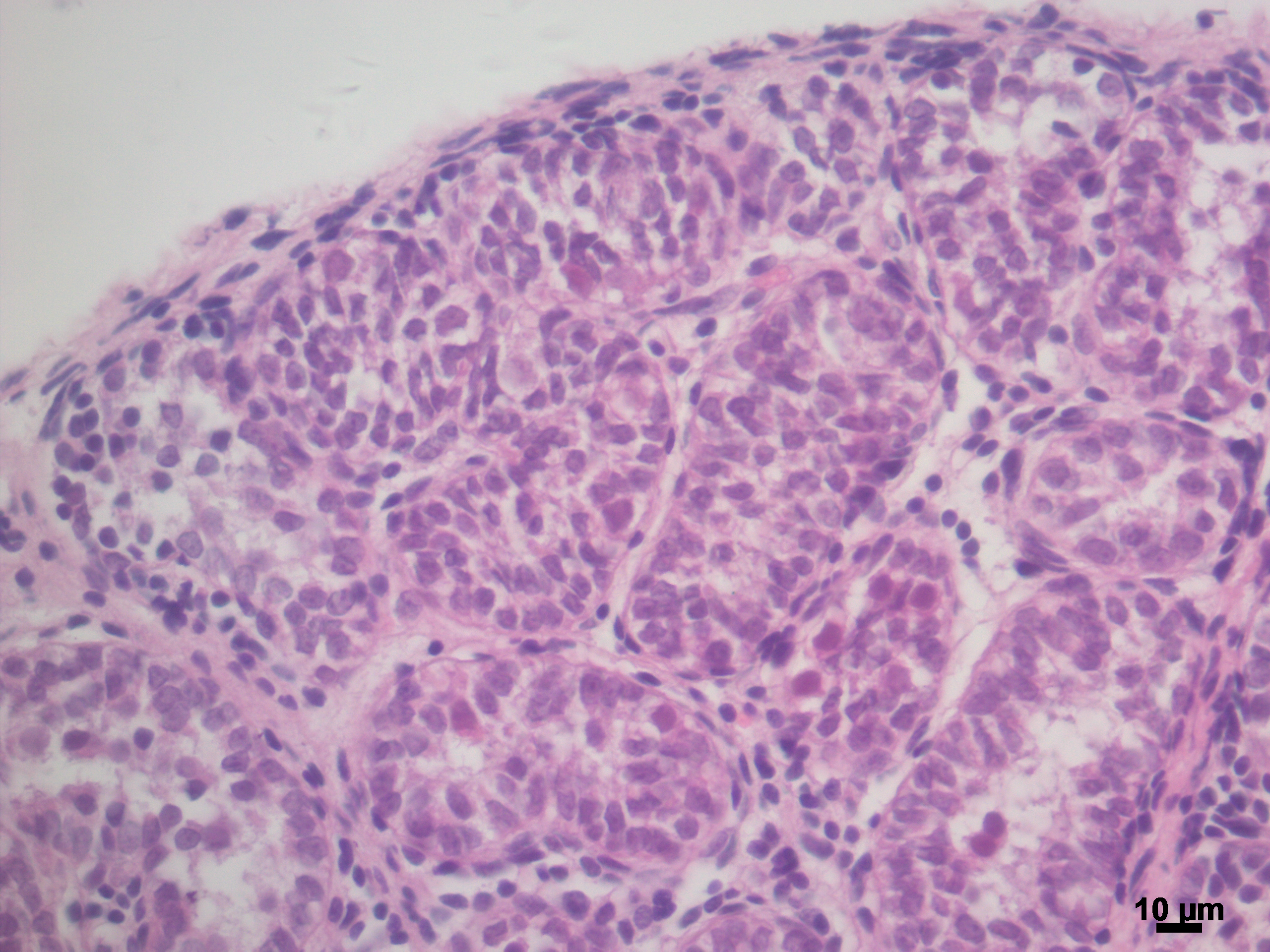

Supplement: Supplementary file 2 — Supplementary Material [file JCMM-26-1567-s001.tif]

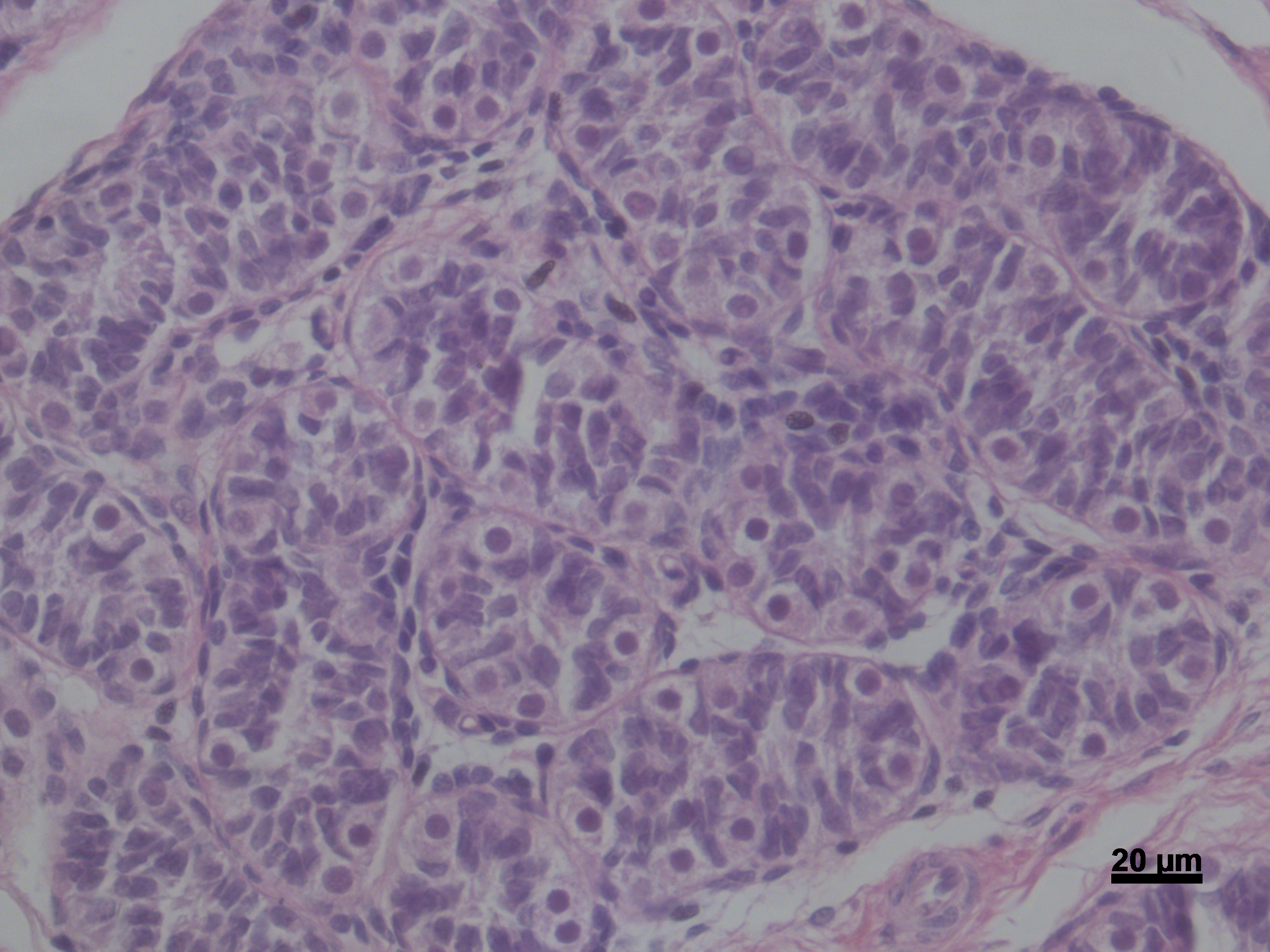

Supplement: Supplementary file 3 — Supplementary Material [file JCMM-26-1567-s004.tif]

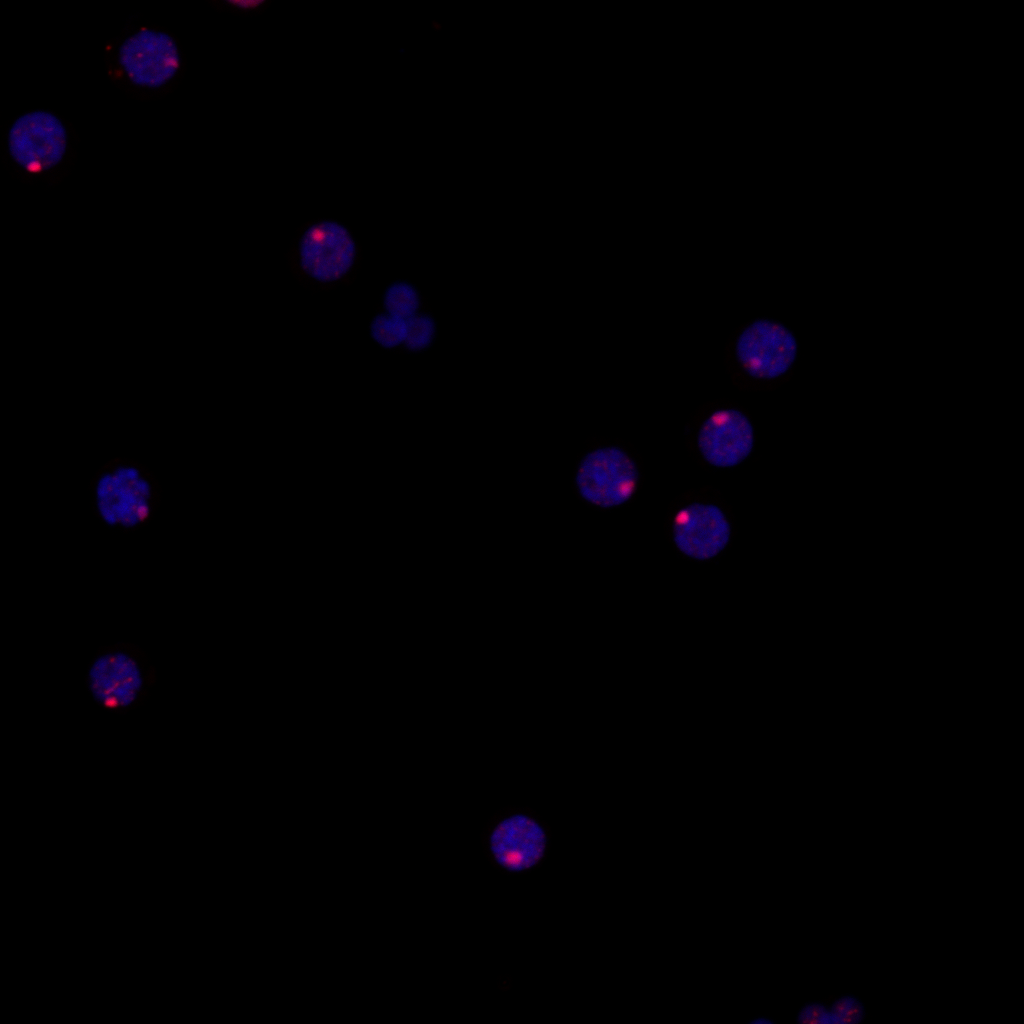

Supplement: Supplementary file 4 — Supplementary Material [file JCMM-26-1567-s003.tif]

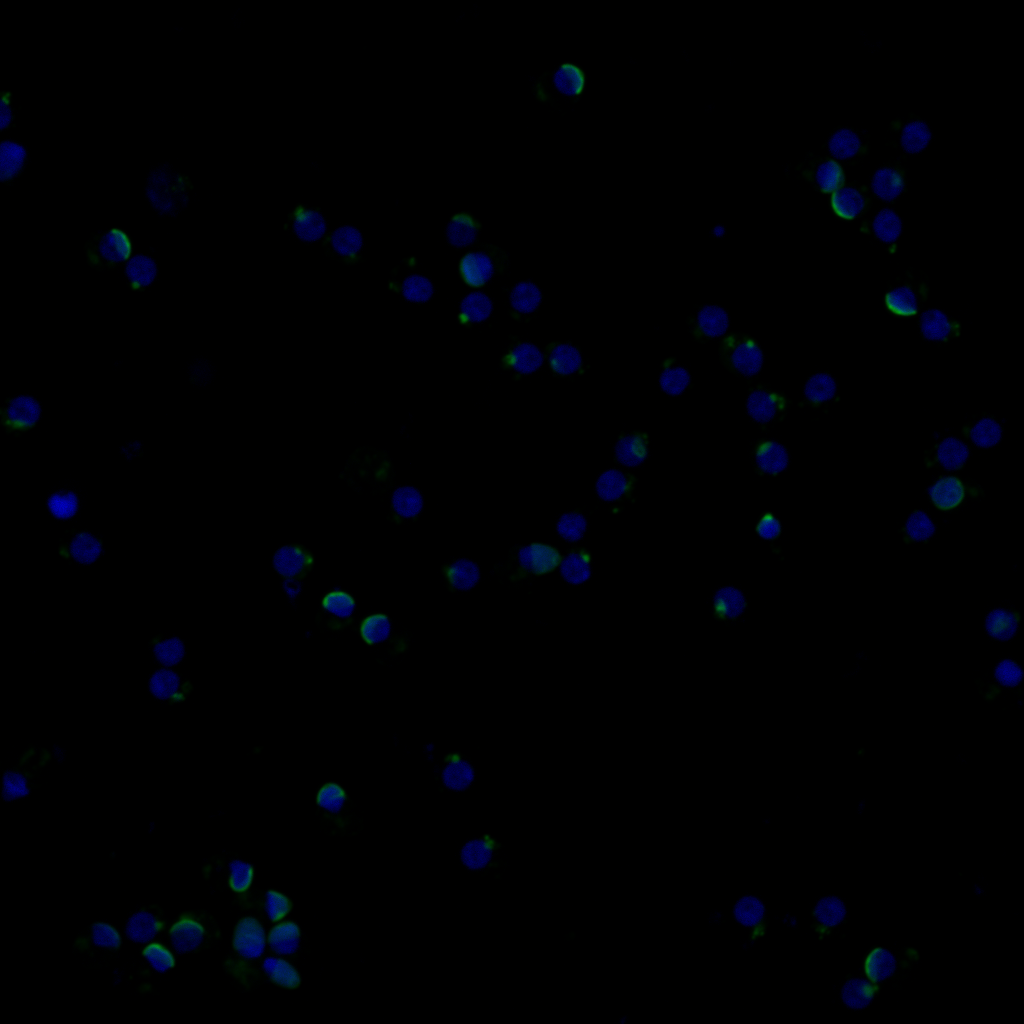

Supplement: Supplementary file 5 — Supplementary Material [file JCMM-26-1567-s008.tif]

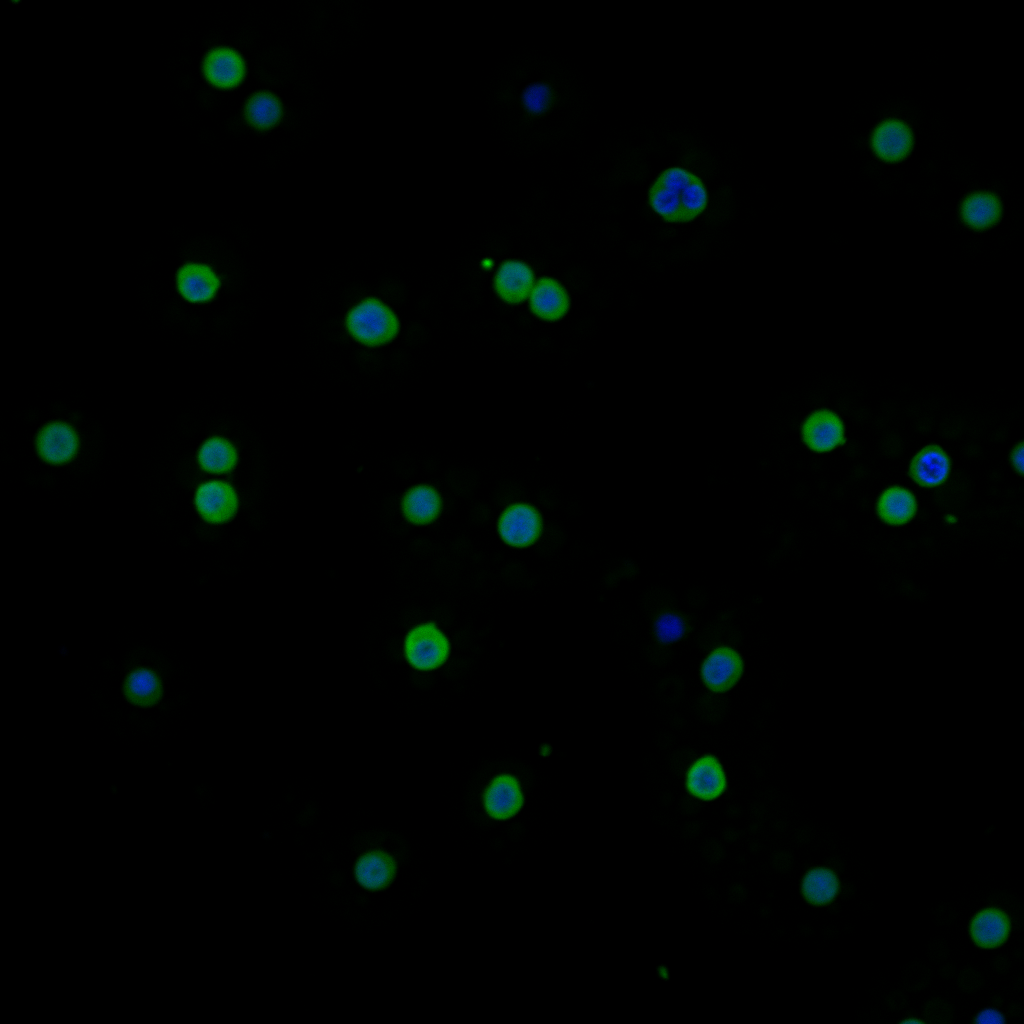

Supplement: Supplementary file 6 — Supplementary Material [file JCMM-26-1567-s009.tif]

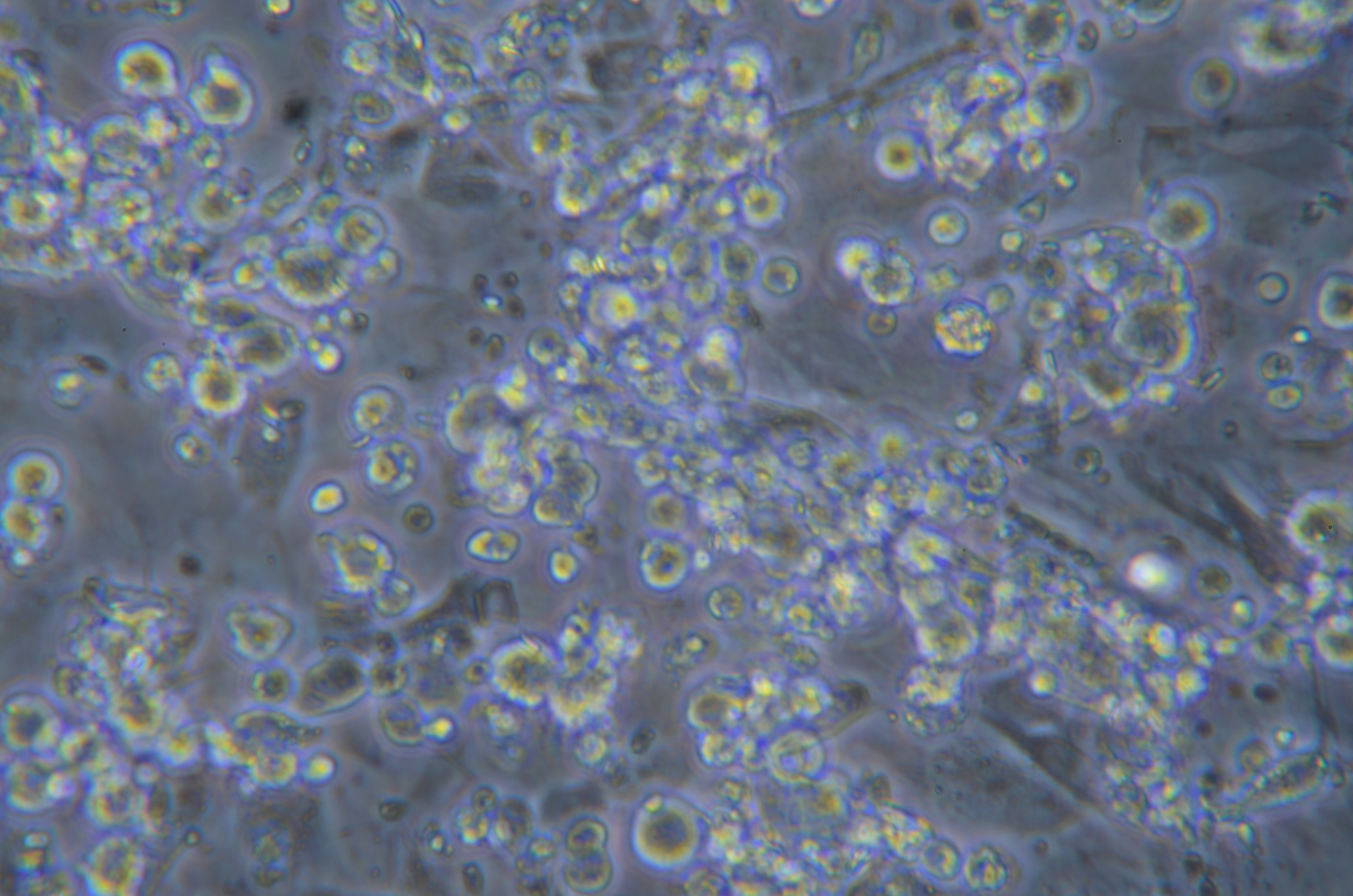

Supplement: Supplementary file 7 — Supplementary Material [file JCMM-26-1567-s006.jpg]

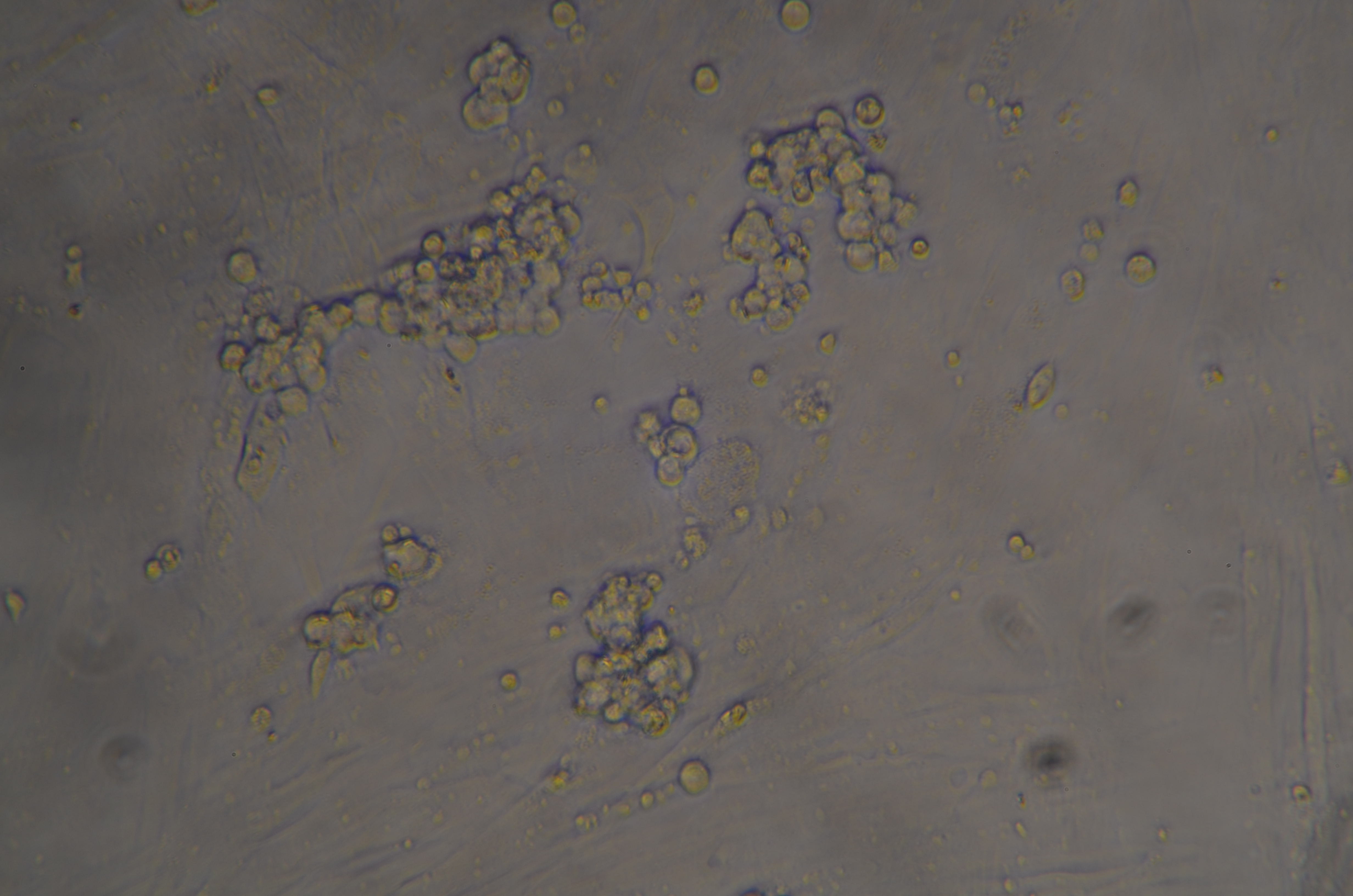

Supplement: Supplementary file 8 — Supplementary Material [file JCMM-26-1567-s002.jpg]

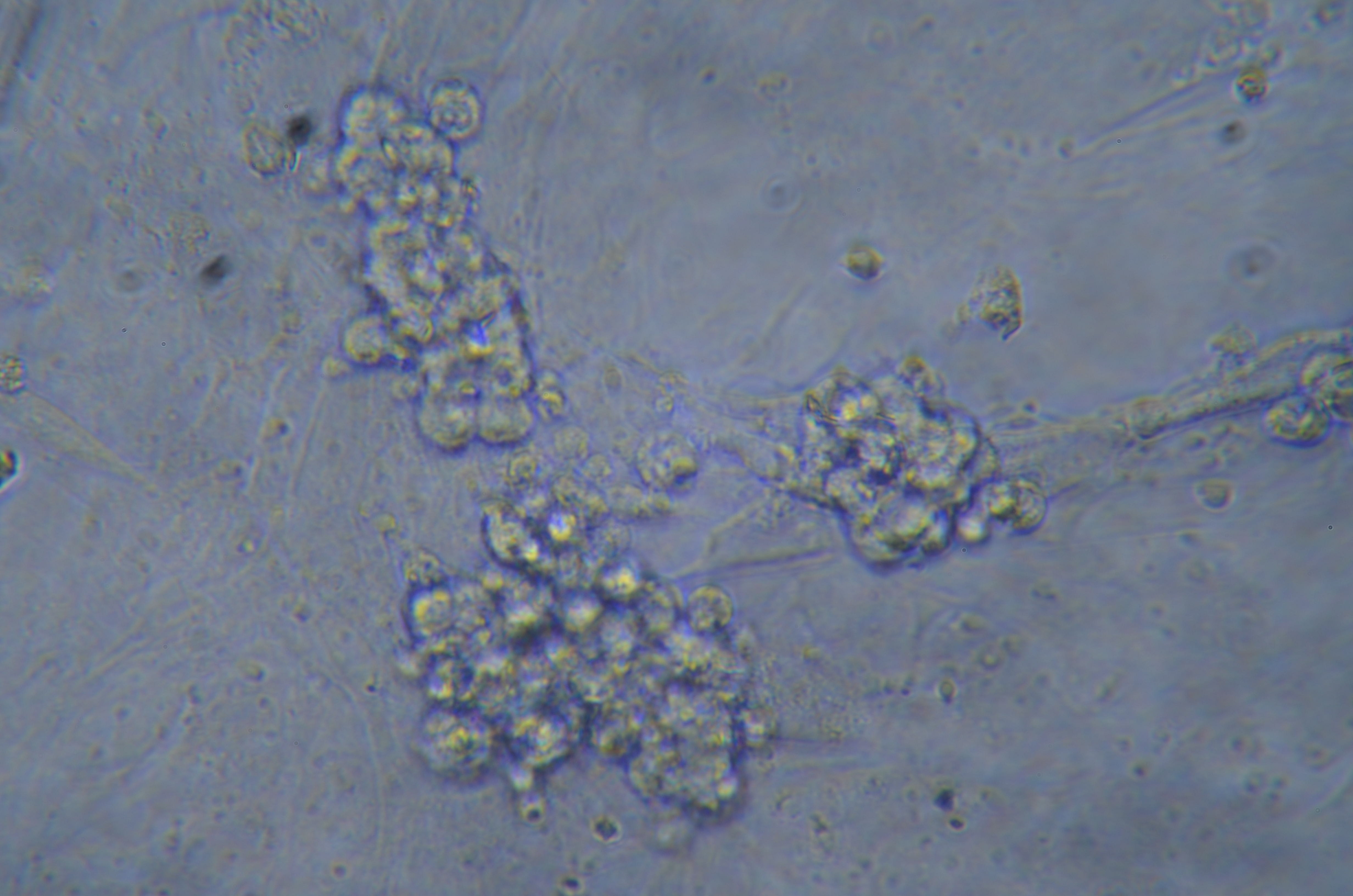

Supplement: Supplementary file 9 — Supplementary Material [file JCMM-26-1567-s007.jpg]

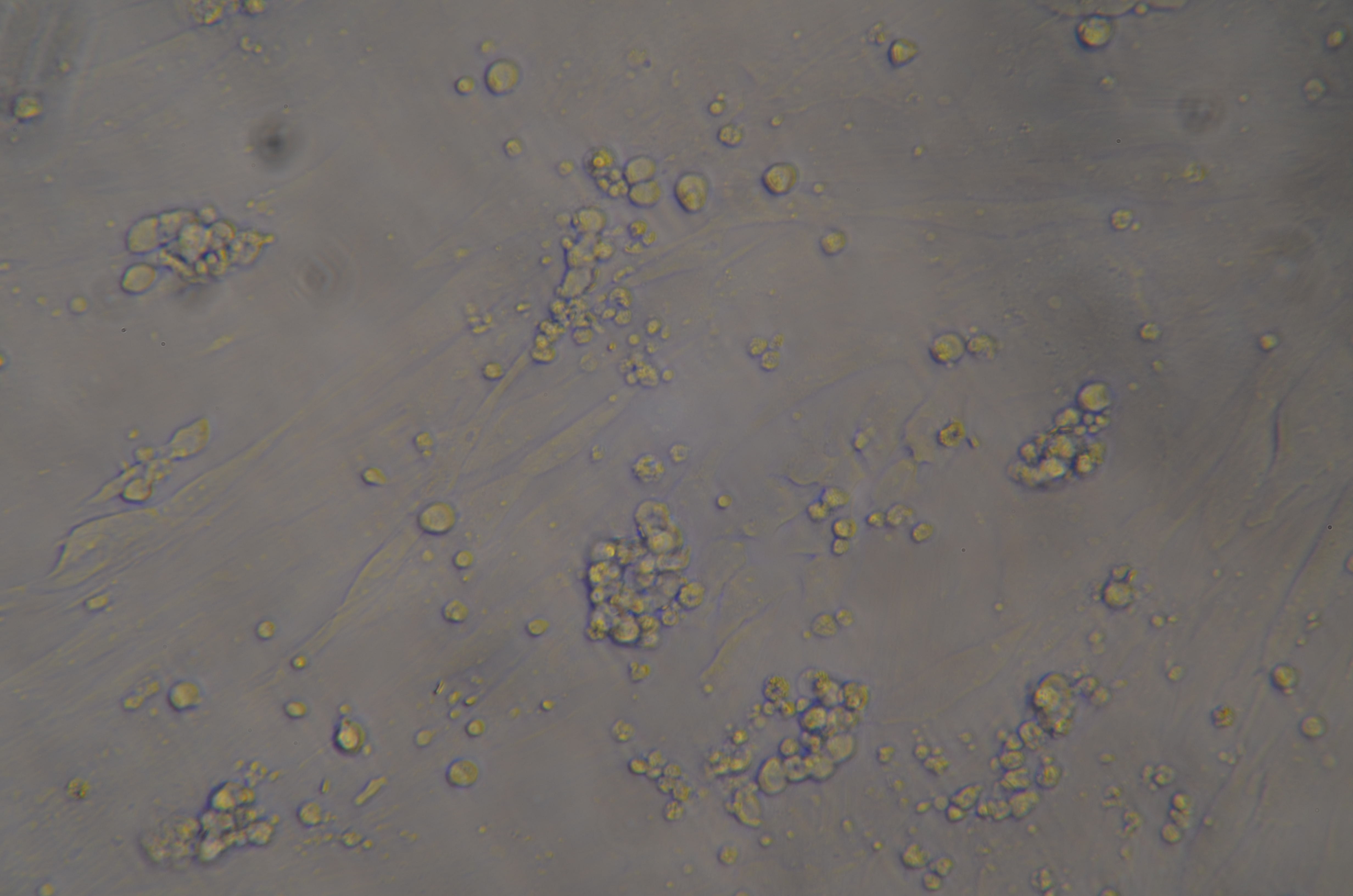

Supplement: Supplementary file 10 — Supplementary Material [file JCMM-26-1567-s010.jpg]
